# Prevalence of endo‐ and ecto‐parasites of equines in Iran: A systematic review

**DOI:** 10.1002/vms3.321

**Published:** 2020-07-09

**Authors:** Faham Khamesipour, Taghi Taktaz‐Hafshejani, Kwenti E. Tebit, Seyed Mostafa Razavi, Seyed Reza Hosseini

**Affiliations:** ^1^ Cellular and Molecular Research Center Sabzevar University of Medical Sciences Sabzevar Iran; ^2^ Department of Pathobiology School of Veterinary Medicine Shiraz University Shiraz Iran; ^3^ Department of Clinical Sciences Faculty of Veterinary Medicine Shahrekord Branch Islamic Azad University Shahrekord Iran; ^4^ Department of Microbiology and Parasitology University of Buea Buea Cameroon; ^5^ Department of Pathobiology Faculty of Veterinary Medicine Shahrekord Branch Islamic Azad University Shahrekord Iran

**Keywords:** ectoparasites, equine, helminths, Iran, parasitic infections, prevalence, protozoa

## Abstract

Equines are subject to infection with many parasites, which threaten their health. In the present study, we systematically reviewed existing literature on the prevalence of endo‐ and ectoparasites of equines in Iran. Major electronic databases, including PubMed, PubMed Central, Google Scholar, Science Direct and Scientific Information Database (SID), were searched (Last updated 11/05/2018) for relevant literature of parasites that have been identified from equines in Iran. Of the 1809 titles produced by bibliographic search, 38 were included in the review. Twenty‐seven of the studies were on horses, six on donkeys, three on both horses and donkeys, and one study was on both horses and mules. Furthermore, 24 of the studies reported infections caused by protozoa, thirteen by helminths, two by ectoparasites, and one by both protozoa and helminths. The overall pooled prevalence of parasitic infection was 28.8% (95%CI: 22.9–35.7, *I* ^2^ = 93.4%). Helminths were the most prevalent parasites 46.7% (95% CI: 24.1–70.7, *I* ^2^ *= *96.0%). Furthermore, donkeys were the most affected equine, with a prevalence of 70.7% (95% CI: 53.2–83.7, *I* ^2^ *= *92.5%). The protozoa frequently reported included nine species belonging to the genera: *Neospora*, Toxoplasma, *Theileria*, *Babesia* and *Eimeria*. Also, the helminths frequently reported included 21 species belonging to the genera: *Strongylus*, *Dicrocoelium*, *Oxyuris*, *Habronema*, *Echinococcus*, *Dictyocaulus*, *Cyathostomum*, *Probstmayria*, *Anoplocephala*, *Setaria* and *Fasciola*. Ticks were the only ectoparasites frequently reported. Parasitic fly species of the genera *Gasterophilus* were also reported. The study‐level risk of bias was likely to be high because of differences in study design. Parasitic infections of equines in Iran are frequent and caused by a diversity of parasites, which threatens the health and well‐being of these animals. Further research is needed in the area to identify the risk factors of infection for effective control of the parasites.

## Graphical abstract

In this study, we systematically reviewed existing literature on the prevalence of endo‐ and ecto‐parasites of equines in Iran.

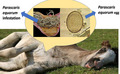

## 1 INTRODUCTION

“Equine” is often used to refer to members of the genus *Equus*, which include horses (*Equus ferus caballus*), donkeys (*Equus africanus asinus*), mules, zebra (*Equus zebra*), etc. There are an estimated 110 million equines in the developing world (Ali & Yagoob, 2015). More than 90% of the estimated 44 million donkeys in the world are in developing countries (Matthee, Krecek, & Milne, 2000). In developing countries, equines contribute greatly to the development of the agricultural economy, being used as a means of transportation due to economic and/or topographical constraints (Ali & Yagoob, 2015; Pritchard, Lindberg, Main, & Whay, 2005), and also are used in recreational activities such as sport, gaming and entertainment.

Equines are often subjected to many diseases which affect their performance (Khamesipour, Dida, Anyona, Razavi, & Rakhshandehroo, 2019; Moazeni, Khamesipour, Anyona, & Dida, 2019; Nejat et al., 2015; Pritchard et al., 2005; Taktaz‐Hafshejani et al., 2015). Among these, parasitic diseases stand out as a major challenge to the health and welfare of horses, especially in developing countries (Pritchard et al., 2005). Parasites can be grouped as ectoparasites (i.e. parasites living on the body surfaces of the host) or endoparasites (i.e. parasites living inside the host), the latter can be further classified as protozoa or helminths (Kwenti, 2017). Ticks, especially the hard ticks (Ixodidae), are the most frequent ectoparasites reported in equines (Davari et al., 2017). Protozoa commonly infecting equines include *Eimeria sp., Neospora sp., Theileria (Babesia) equi, Babesia caballi, Cryptosporidium sp*. and *Toxoplasma gondii* (Foster, 1942). Helminths commonly infecting equines include *Trichostrongylus sp.,* Paramphistommatidae*, Fasciola sp., Strongylus sp., Dicrocoelium sp., Moniezia sp., Trichuris sp., Oxyuris sp., Parascaris sp., Prostmayaria sp., Strongyloides sp*. and the Cyathostomins (Hosseini et al., 2009). Helminths, notably the gastrointestinal parasites, have been recognized as one of the most critical problems of equines in developing countries (Perry, Randolph, McDermott, Sones, & Thornton, 2002) and infection rates have been estimated to be as high as 90% in equines (Fikru, Reta, Teshale, & Bizunesh, 2005; Valdez‐Cruz, Hernandez‐Gil, Galindo‐Rodriguez, & Alonso‐Diza, 2006). It has been estimated that over 80% of donkeys in an area can be infected (Burden, du Toit, Hernandez‐Gil, Prado‐Ortiz, & Trawford, 2010; duToit, Burden, & Dixon, 2008; Getachew, Trawford, Feseha, & Reid, 2010). Studies of parasitic infection in equines have uncovered a diversity of helminth species (Hosseini et al., 2009; Trawford & Getachew, 2008). Nearly all equines have internal parasites, and if left untreated, these parasites can deprive the animal of precious blood nutrients and energy, thereby affecting their performance. Parasites mainly affect the digestive system of equines; however, the respiratory system and other organs may also be affected (Al‐Qudari, Al‐Ghamdi, & Al‐Jabr, 2015). The consequences of parasitic infection in equine may range from diarrhea, anemia, fever, colic, weight loss, weak growth, emaciation, impaired growth, increased susceptibility to other infectious diseases and sudden death (Arfaei et al., 2013; Taylor, Coop, & Wall, 2007).

In Iran, there are over two million equids, of which about 75% are donkeys (Hosseini et al., 2009). Like in other parts of the world, equines contribute to the agricultural economy of Iran and are a valuable means of transportation in some areas of the country. Parasites are also a menace to the health and welfare of equines, but a systematic review of the parasite status of equine in Iran is not readily available. The objective of the present study was to systematically review the existing literature on the prevalence and aetiology of parasitic diseases affecting equines in Iran to inform control policies.

## 2 MATERIALS AND METHODS

A literature review was carried out between 1st of April and 11th of May 2018, to identify scientific articles reporting parasitic infections of equines in Iran. The current study conforms to the Preferred Reporting Items for Systematic Reviews and Meta‐Analysis (PRISMA) guidelines (Moher et al. 2009) (File S1).

### 2.1 Search strategy and selection criteria

Relevant studies were searched in electronic databases, including PubMed, PubMed Central, Google Scholar, ScienceDirect and Scientific Information Database (SID) using the keywords: Parasites OR Infection OR Equine OR Horse OR Donkey OR Mule OR Iran.

**FIGURE 1:**
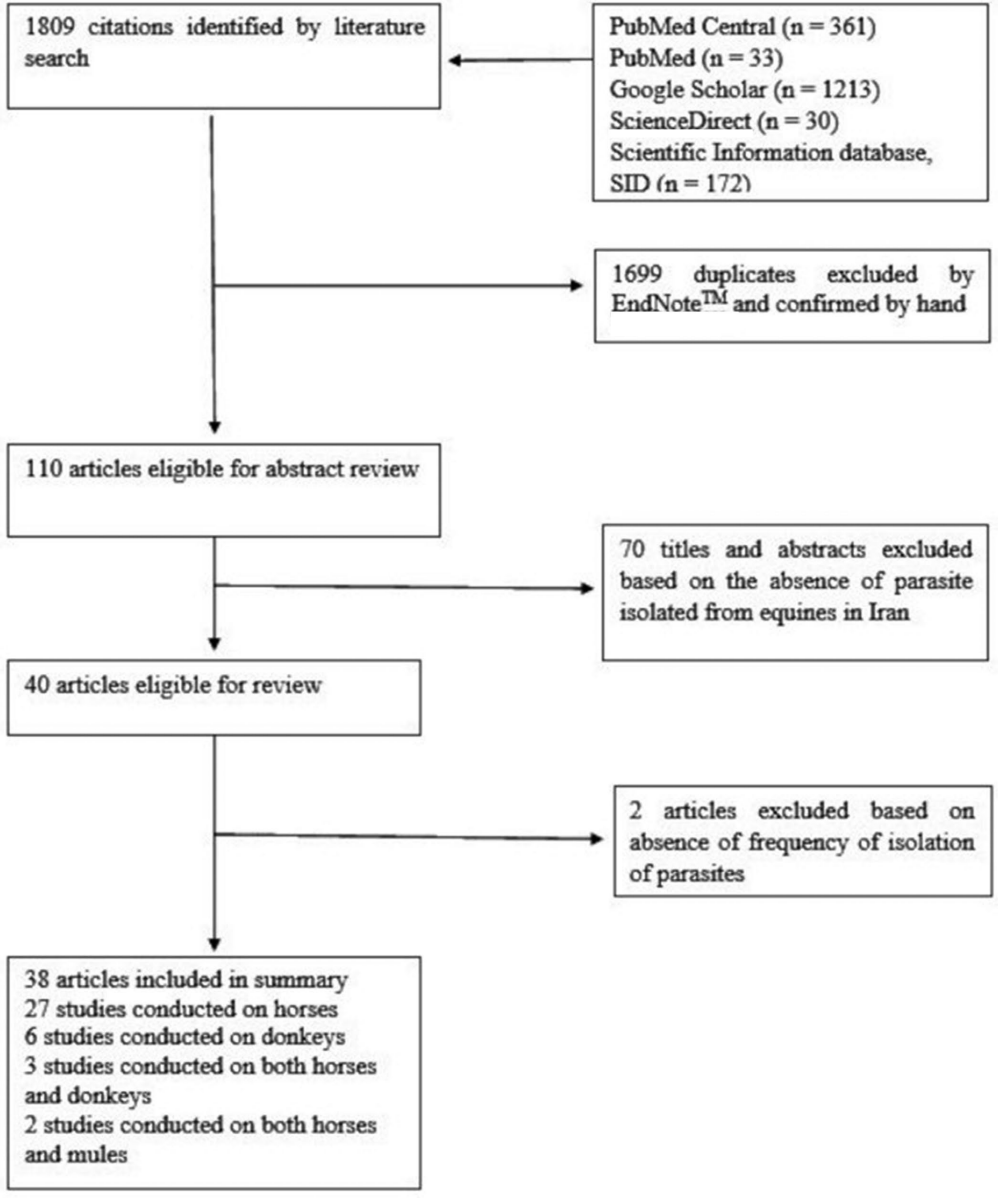
Flowchart of the selection process for publications included in this review

No time limits were defined, and articles reporting parasitic infections of equine irrespective of the methods used for identification (i.e. serology, coprology or molecular methods) were selected. Subsequently, the titles and abstracts of the selected articles were examined by two reviewers independently (parallel method), to identify articles reporting parasitic infections in equines in Iran. Where there was any discrepancy in their report, a third reviewer was brought in to resolve it. Relevant papers were also manually cross‐checked to identify further references. In the articles selected, the following data were extracted by one reviewer and crosschecked by a second: Type of parasitic infection, the prevalence of infection, species of parasites identified and their frequencies, host type involved (horses, donkeys, mules, etc.), the geographical location of study, association with host factors (age, sex or season) and the method used to identify the parasite. Articles were excluded when they did not report any parasite species. The selection process is detailed in Figure 1.

### 2.2 Statistical analysis

Pooled prevalence was determined using Comprehensive Meta‐Analysis V3.3.070 software (Biostat, USA). Data were pooled using a Fixed and random‐effect model. The heterogeneity between these studies was assessed with the *I* ^2^ test. An *I* ^2^ value of >50% indicated substantial heterogeneity. For the pooling of the results, a more conservative random‐effect model was used as heterogeneity was substantial.

## 3 RESULTS

The results revealed publications from 2005 to 2017. The review of the literature provided 1809 titles (361 on PubMed Central, 33 on PubMed, 1213 on Google Scholar, 30 on ScienceDirect and 172 on SID), 1699 of which were discarded as they were found to be duplicated using a reference manager software (EndNoteTM) and confirmed manually (Figure 1). During the review of the remaining 110 works, 70 abstracts were discarded because they did not contain information on the parasites detected. The remaining 40 studies were analysed, rejecting two articles that were not written in English and did not contain an abstract in English (Figure 1).

A total of 38 articles were selected, all written in English. Twenty‐seven of the studies were conducted on horses (71.1%, 95% CI [54.1–84.6]), six (15.8%, 95% CI [6.0–31.3]) conducted on donkeys, three (7.9%, 95% CI [1.7–21.4]) conducted on both horses and donkeys and two (5.3%, 95% CI [0.6–17.8]) conducted on both horses and mules.

Fifteen studies reported intestinal parasites 15 (39.5%, 95% CI [24.0–56.6]), 20 (52.6%, 95% CI [35.8–69.0]) reported blood parasites, three (7.9%, 95% CI [1.7–21.4]) reported tissue parasites. Twenty‐four studies reported protozoa 24 (63.2%, 95% CI [46.0–78.2]), 13 (34.2%, 95% CI [19.6–51.4]) reported helminths, two (5.3%, 95% CI [0.6–17.8]) reported ectoparasites and one (2.6%, 95% CI [0.07–14.8]) reported both helminth and protozoa parasites.

Five of the studies were performed in the Northern (13.2%, 95% CI [4.4–298.1]), 11 (29.0%, 95% CI [15.4–45.9]) in the Northeastern, 10 (26.3%, 95% CI [13.4–43.1]) in the Northwestern, three (7.9%, 95% CI [1.7–21.4]) in Western, one (2.6%, 95% CI [0.07–14.8]) in the Southern, and eight (21.1%, 95% CI [9.6–37.3]) in the Southwestern regions of Iran.

**FIGURE 2:**
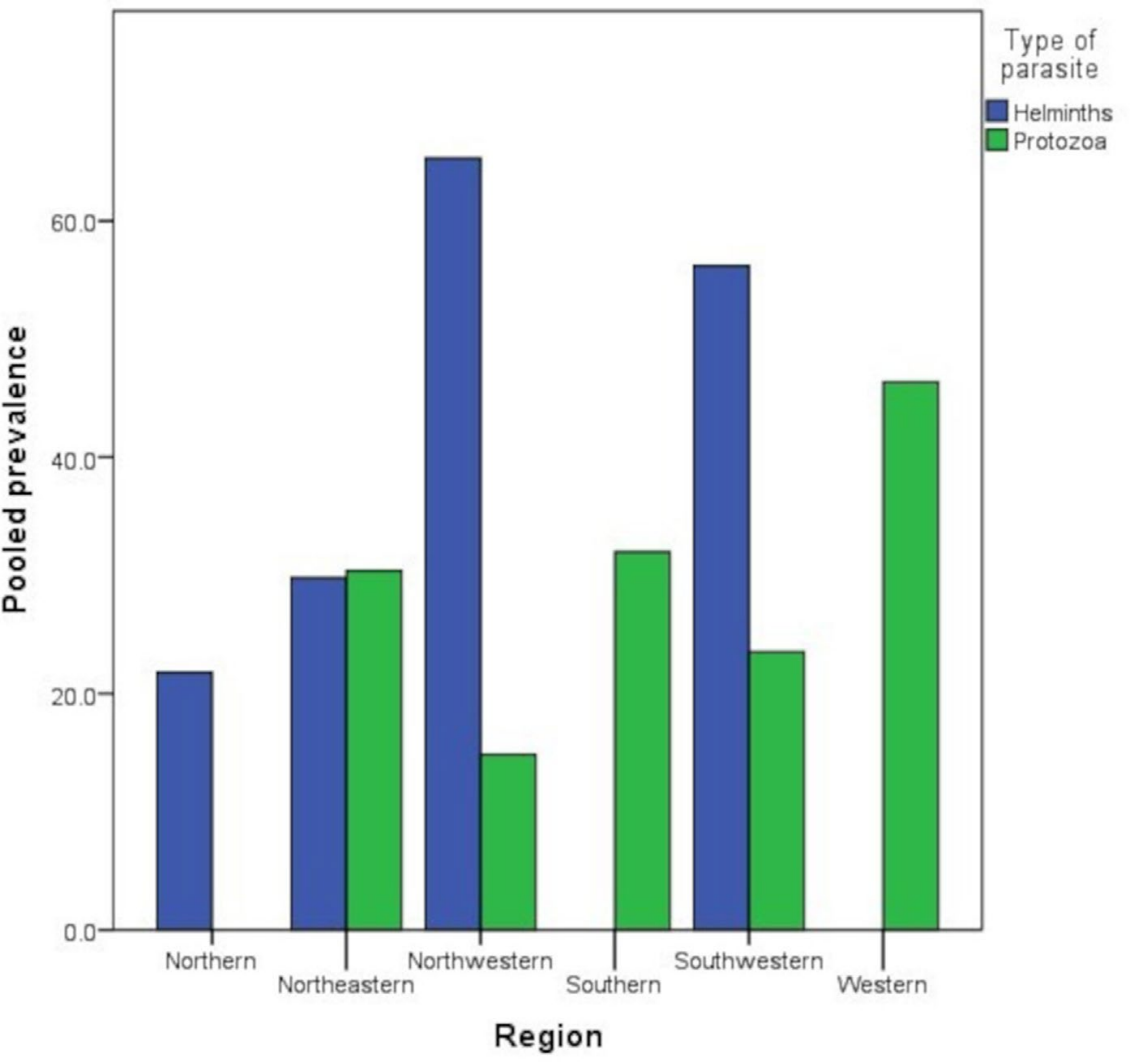
Pooled prevalence of parasitic infection by region

Overall, the prevalence of parasitic infection in equine varied between 1.72% and 96.77% (Table 1). The pooled prevalence was 28.8% (95%CI: 22.9–35.7, *I* ^2^ = 93.4%). The pooled prevalence of protozoa, helminth and ectoparasite (ticks were the only species identified) was 26.2% (95% CI: 20.06–32.7, *I* ^2^ *= *91.9%), 46.7% (95% CI: 24.1–70.7, *I* ^2^ *= *96.0%) and 14.8% (95% CI: 9.5–22.2, *I* ^2^ *= *0.0%), respectively. The prevalence of parasitic infection was higher in donkeys 70.7% (95% CI: 53.2–83.7, *I* ^2^ *= *92.5%) compared to horses 23.4% (95% CI: 18.3–29.4, *I* ^2^ *= *92.3%) or mule 12.5%. Furthermore, the prevalence of helminthic infection was highest in the northwestern region meanwhile prevalence of protozoa infection was highest in the western region of the country (Figure 2). Generally, the helminth parasite species reported were very diverse compared to protozoa parasites (21 species versus nine species).

**TABLE 1:** Summary of the prevalence of parasitic infections affecting equines in Iran

Parasitic infection/disease		Aetiology	Method of detection	Site of isolation	Host type affected	Prevalence (%)	Reference
Protozoa							
1	Neosporosis	*Neospora caninum, Neospora hughesi*	Serology	Tissues	Horse	20–40.8	(Gharekhani & Heidari, 2014; Gharekhani et al., 2013; Hosseini et al., 2011; Moraveji et al., 2011; Tavalla et al., 2015; Yagoob, 2011)
					Donkey	52	(Gharekhani et al., 2013)
2	Toxoplasmosis	*Toxoplasma gondii*	Serology	Tissues	Horse	11.5–71.2	(Hajialilo, Ziaali, Harandi, Saraei, & Hajialilo, 2010; Raeghi et al., 2011; Razmi et al., 2016; Tavalla et al., 2015)
3	Cryptosporidiosis	*Cryptosporidium parvum, C. hominis, C. felis*	Faecal, molecular		Horse	10.56–26.66	(Ghadrdan‐Mashhadi et al., 2013; Naghibi & Vahedi, 2002; Rasuli et al., 2012; Tavassoli et al., 2007)
				Tissues, GIT	Mule	12.5	(Rasuli et al., 2012)
4	Equine coccidiosis	*Eimeria leuckarti*	Faecal	GIT	Horse	7.68	(Ghahfarrokhi et al., 2014)
					Donkey	7.68	(Ghahfarrokhi et al., 2014)
5	Equine piroplasmosis	*Theileria (Babesia) equi, Babesia caballi*	Blood smears and molecular	Tissues	Donkey	50.94	(Abedi et al., 2015)
					Horse	4.1–96.77	(Arfaei et al., 2013; Davoodi et al., 2010; Habibi et al., 2016; Hassanpour & Nematollahi, 2014; Hosseini, Taktaz‐Hafshejani, & Khamesipour, 2017; Malekifard et al., 2014; Sakha, 2007)
Nematode							
6	Oxyurosis	*Oxyuris equi*	Faecal, necropsy	GIT	Horse	3.84–26	(Eslami et al., 2005; Ghahfarrokhi et al., 2014; Hosseini et al., 2009; Hossien et al., 2008; Khosravi et al., 2012)
					Donkey	11.53	(Ghahfarrokhi et al., 2014)
7	Strongylosis	*Strongylus vulgaris, Strongylus equinus, Strongylus edentates,*	Faecal, necropsy		Horse	28.3–34	(Ali & Yagoob, 2015; Eslami et al., 2005; Hossien et al., 2008; Khosravi et al., 2012)
				GIT	Donkey	100	(Borji, Moosavi, & Ahmadi, 2014; Hosseini et al., 2009; Tavassoli et al., 2016)
8	Cyathostominosis	*Cyathostomum pathatum, cylicocyclus elongates, Cylicostephanus longibarsatus, Cylicostephanus goldi, Cylicocyclus nassatus*	Faecal, necropsy	GIT	Donkey	53.3	(Hosseini et al., 2009; Oryan, Kish, & Rajabloo, 2015)
					Horse	4–22	(Ali & Yagoob, 2015)
9	Parascariosis	*Parascaris equorum,*	Faecal, necropsy	GIT	Horse	10–44	(Eslami et al., 2005; Ghahfarrokhi et al., 2014; Hossien et al., 2008; Khosravi et al., 2012)
					Donkey	3.84–20	(Hosseini et al., 2009; Ghahfarrokhi et al., 2014; Tavassoli et al., 2016]
10	Summer Sores (Cutaneous Habronemosis)	*Habronema muscae, Habronema majus, Draschia (Habronema) megastoma*	Faecal, necropsy	GIT	Donkey	1.72–66.6	(Hosseini et al., 2009; Tavassoli et al., 2016)
11	Equine hydatidosis or echinococcosis	*Echinococcus granulosus*	Necropsy	Liver	Horse, donkey	3.11	(Eslami, Shayan, & Bokaei, 2014; Sakhaee, Golchin, Amiri, Fayed, & Eydi, 2016)
12	Lungworm infection	*Dictyocaulus arnfieldi*	Faecal	GIT	Horse	‐	(Sharifi, Borji, & Milani, 2010)
13	Probstmayriosis	*Probstmayria vivipavra*	Necropsy	GIT, Liver	Donkey	20	(Hosseini et al., 2009)
14	Trichostrongylosis	*Trichostrongylus axei*	Necropsy	GIT, Liver	Donkey	6.6	(Hosseini et al., 2009)
15	Filariosis	*Setaria equina*	Necropsy	GIT, Liver	Donkey	6.6	(Hosseini et al., 2009)
16	Equine parafilariosis	*Parafilaria multipapillosa*	Blood smears	Tissues	Horse and Donkey	1.4–41.3	(Maloufi, 1995)
Trematode							
17	Dicrocoeliosis	*Dicrocoelium denderiticum*	Faecal, necropsy	Intestines	Horse	17.14–56	(Khosravi et al., 2012)
					Donkey	6.6	(Hosseini et al., 2009)
18	Fasciolosis	*Fasciola hepatica*	Necropsy	GIT, Liver	Donkey	6.6	(Hosseini et al., 2009)
Cestode							
19	Anoplocephalosis	*Anoplocephala perfoliata*	Necropsy	GIT, Liver	Donkey	12.3	(Hosseini et al., 2009)
Ascari							
20	Tick infestation	*Hyalomma spp., Rhipicephalus spp., Boophilus spp*.	Hand‐picking	Body surfaces	Horse	16.45–52	(Davoodi et al., 2010; Khosravi et al., 2012)
Insect (Parasitic fly)							
21	Gasterophilosis	*Gasterophilus intestinalis, G. nasalis, G. inermis*	Necropsy	GIT, liver	Horse, Donkey, Mule	16.07–66.6	(Hosseini et al., 2009; Mashayekhi & Ashtari, 2013; Tavassoli & Bakht, 2012)

## 4 DISCUSSION

This review revealed a generally high prevalence of parasitic infections in equine in Iran. Among the parasites infecting equines in Iran, the helminth parasites, especially the nematodes, were the most frequent (up to 100%) and diverse group of parasites (Table 1). A majority of the parasites reported in this study were observed to infect the gastrointestinal tract (GIT). Gastrointestinal parasitism is known to be acquired passively (i.e., through the ingestion of infective larvae on pasture). However, in some species, larvae burrow through the skin or are transmitted by invertebrate intermediate hosts (Anderson, 2000). Ticks were the only ectoparasite frequently isolated from equines in Iran. Ticks are non‐permanent obligate and the most frequent ectoparasites of terrestrial vertebrates constituting a serious threat to animal and human health in many parts of the world. They are capable of exerting direct damage as well as act as vectors of many parasitic, viral, and bacterial pathogens (De la Fuente, Estrada‐Pena, Venzal, Kocan, & Sonenshine, 2008; Allan 2001). From this review, one notable equine parasitic disease transmitted by ticks in Iran was piroplasmosis caused by *Theileria equi* and *Babesia caballi* (Abedi, Razmi, Seifi, & Naghibi, 2015; Arfaei et al., 2013; Davoodi, Rauli, & Jafari, 2010; Habibi et al., 2016; Hassanpour & Nematollahi, 2014; Malekifard, Tavassoli, Yakhchali, & Darvishzadeh, 2014; Sakha, 2007). Control of ticks and tick‐borne diseases of equine is therefore vital for the protection of the health of the animals and an increase in their productivity in the area. Control of parasites of animals is equally important in protecting human health as some of these parasites are zoonotic. At least one of the studies reviewed showed a higher rate of *Cryptosporidium* infection in persons who were in contact with infected animals (Naghibi & Vahedi, 2002). Another study in France reports of three cases of acquired toxoplasmosis in humans caused by the consumption of raw horse meat (Pomares et al., 2011). Although this does not fit in the traditional classification of parasites (as either ectoparasite or endoparasite), the larvae of parasitic flies, *Gasterophilus intestinalis, G. nasalis, G. inermis*, have also been reported to cause serious health problems to equines in Iran (Davari et al., 2017; Hosseini et al., 2009; Tavassoli & Bakht, 2012).

The parasitic infections frequently reported from this review included neosporosis, equine piroplasmosis, and strongylosis. Neosporosis is caused by *Neospora caninum*, an Apicomplexan protozoan parasite with a worldwide distribution (Hosseini et al., 2011). The parasites can infect a wide range of animal species, including cattle, sheep, goats, horses, dogs, and cats, and have been associated with abortion, protozoal myeloencephalitis, and neuromuscular disorder signs in equine (Finno, Aleman, & Pusterla, 2007). Equine piroplasmosis is a haemolytic disease caused by two intra‐erythrocytic hemo‐protozoan, *Theileria equi* and *Babesia caballi* (Mahmoud et al., 2016). The disease is characterized by fever, anaemia, red urine, jaundice, oedema, weight loss and even death in equine (Mahmoud et al., 2016). On the other hand, strongylosis is caused by several nematodes, often referred to as the small and large strongyles (Tavassoli, Yamchi, & Hajipour, 2016). They are frequently responsible not only for poor health, but also for gastrointestinal dysfunction, including colic, and infection with some such as acute larval cyathostomosis may be fatal (Love, Murphy, & Mellor, 1999).

Reports of vector‐borne parasitic diseases such as filariosis (Lia et al., 2017; Radwan, Ahmed, Elakabawy, Ramadan, & Elmadawy, 2016) and trypanosomosis (Luckins, 1994), known to cause major problems in equines worldwide, have not been reported in Iran. The only filarial species that has been reported in equines in Iran are *Setaria equina* (Hosseini et al., 2009) and *Parafilaria multipapillosa* (Maloufi 1995). The under‐reporting of filarial parasites in equines in the country may be due to individual study level biases in the design of the different studies, pertaining to the methods used to detect the presence of parasites; serology was used to detect exposure to most of the protozoa parasites meanwhile concentration techniques, culture and molecular methods were not used in all the studies, constituting a major limitation to the study. At least one study has reported a higher detection rate of parasitic infection using molecular methods compared with serological and standard parasitological techniques (Habibi et al., 2016; Mahmoud et al., 2016). The differences in the diagnostic methods may also explain the variability in the observed prevalence of the parasitic infections from one area to another.

This study demonstrates heterogeneity in the distribution of parasitic infection in Iran. The pooled prevalence of protozoa infection was highest in the western region meanwhile, the prevalence of helminth infection was highest in the northwestern region. The discrepancy in the prevalence of parasitic infection in the different areas of Iran could also be attributed to the inter‐regional differences in the endemicity of the parasites. Climatic and cultural differences may also be a contributing factor to these inter‐regional differences.

This review also revealed that most (but not all [Eslami, Bokai, & Tabatabai, 2005; Hossien, Bokaei, & Roudgari, 2008; Khedri, Radfar, Borji, & Azizzadeh, 2014; Razmi, Abedi, & Yaghfoori, 2016]) of the studies failed to observed a significant association between prevalence of parasites and age of the animals (Armand, Solhjoo, Shabani‐Kordshooli, Davami, & Sadeghi, 2016; Eslami et al., 2005; Ghadrdan‐Mashhadi, Hamidienjat, & Alizadehnia, 2013; Gharekhani, Tavoosidana, & Naderisefat, 2013; Malekifard et al., 2014; Tajik, Mirshahi, Razmi, & Mohammadi, 2010; Tavalla et al., 2015; Tavassoli, Sodagar‐Skandarabadi, & Soltanalinejad, 2007). Also, most (but not all [Khedri et al., 2014]) of the studies failed to observe any significant association between the prevalence of parasites and sex (Hossien et al., 2008; Hosseini et al., 2009; Hosseini et al., 2011; Raeghi, Akaberi, & Sedeghi, 2011; Rasuli, Khodadadi, Sadagiyani, Moradpoor, & Salmanzadeh, 2012; Gharekhani et al., 2013; Ghahfarrokhi, Ahmadi, Shahraki, & Azizi, 2014; Hassanpour & Nematollahi 2014; Mcallister, 2014; Tavassoli et al., 2016). And lastly, most (but not all [Hossien et al., 2008; Khedri et al., 2014]) of the studies failed to observe any significant association between the prevalence of parasites and season (Armand et al., 2016; Tavassoli et al., 2007).

As evident from this review, no study has been conducted to determine risk factors for parasitic infections of equines in Iran, which therefore presents a major challenge for the successful implementation of control strategies in the area. There is, therefore, a need for more empirical research to establish risk factors associated with parasitic infections to develop appropriate control strategies for parasites in equines in Iran.

Control of parasitic infections of equines and other livestock can be achieved using chemical and biological control methods (Kwenti, 2017). Many biological products are available in the markets that have a proven track record to effectively reduce parasite infections in livestock, including the nematopathogenic fungi (*Duddingtonia flagrans*) (Kwenti, 2017), which make a more suitable alternative to the chemical methods. For example, feeding or field trials in sheep have shown that dosing with a few hundred thousand spores per kilogram of live birth weight of *D. flagrans* not only reduced the number of infective larvae but also increased the birth weight of lambs compared with controls (Larsen, 2006).

Furthermore, many vaccines have been developed against parasites of livestock, including vaccines against *Eimeria spp, Theileria spp., Toxoplasma gondii, Babesia spp., Neospora spp*. etc. (Mcallister, 2014; Sharma, Singh, & Shyma, 2015). Vaccines might present a cheaper and more effective alternative to control parasite infection, thereby improving animal production. However, more research is required to develop and evaluate more effective vaccines against parasites.

In conclusion, our work revealed that parasite infections and infestations of equines in Iran are frequent and caused by a diversity of parasites (ectoparasites, protozoa, helminths and parasitic flies), which threatens the health and welfare of the animals. Further research is needed in the area to identify the risk factors of infection for effective control of the parasites.

## CONFLICT OF INTEREST

The authors declare that they have no competing interests.

## AUTHORS’ CONTRIBUTIONS

All authors have read and approved the final version of the paper.

## AUTHOR CONTRIBUTION

**Faham Khamesipour:** Conceptualization; Data curation; Formal analysis; Investigation; Methodology; Project administration; Resources; Supervision; Validation; Visualization; Writing‐original draft; Writing‐review & editing. **Taghi Taktaz Hafshejani:** Validation; Visualization; Writing‐review & editing. **Kwenti Emmanuel Tebit:** Data curation; Formal analysis; Investigation; Methodology; Resources; Software; Validation; Writing‐review & editing. **Seyed Mostafa Razavi:** Resources; Validation; Writing‐review & editing. **Seyed Reza Hosseini:** Investigation; Resources; Writing‐review & editing.

### PEER REVIEW

The peer review history for this article is available at https://publons.com/publon/10.1002/vms3.321.

## Data Availability

The original research articles included in this systematic review are publicly available.
