# Corrigendum

**DOI:** 10.1002/vms3.504

**Published:** 2021-06-18

The following errors were identified in the published article by Khamesipour et al., (2021):

On page 26, left column, second paragraph, lines 16–19 read:

“Helminths commonly infecting equines include *Trichostrongylus* sp., Paramphistommatidae, *Fasciola* sp., *Strongylus* sp., *Dicrocoelium* sp., *Moniezia* sp., *Trichuris* sp., *Oxyuris* sp., *Parascaris* sp., *Prostmayaria* sp., *Strongyloides* sp. and the Cyathostomins (Hosseini et al., 2009)”.

This should be:

“Helminths that have been reported to infect equines include *Trichostrongylus* sp., *Fasciola* sp., *Strongylus* sp., *Dicrocoelium* sp., *Oxyuris* sp., *Parascaris* sp., *Prostmayaria* sp., *Strongyloides* sp., and the Cyathostomins (Hosseini et al., 2009)”.

On page 27, right column, line 1 of the Results section reads:

“The results revealed publications from 2005 to 2017.”

This should be:

“The results revealed publications from 1995 to 2017.”

On pages 28 and 29, Table 1 included some incorrect information. The correct table appears on the next pages.

The authors would like to apologize for the inconvenience caused.

**TABLE 1:** Summary of the prevalence of parasitic infections affecting equines in Iran

Parasitic infection/disease		Etiology	Method of detection	Site of isolation	Host type affected	Prevalence (%)	Reference
Protozoa							
1	Neosporosis	*Neospora caninum, Neospora hughesi*	Serology		Horse	20–40.8	(Hosseini et al., 2011; Yagoob, 2011; Moraveji et al., 2011; Gharekhani et al., 2013; Gharekhani and Heidari, 2014; Tavalla et al., 2015)
					Donkey	52	(Gharekhani et al., 2013)
2	Toxoplasmosis	*Toxoplasma gondii*	Serology	Tissues, GIT	Horse	11.5–71.2	(Hajialilo et al., 2010; Raeghi et al., 2011; Tavalla et al., 2015; Razmi et al., 2016)
3	Cryptosporidiosis	*Cryptosporidium parvum, C. hominis, C. felis*	Fecal		Horse	10.56–26.66	(Naghibi and Vahedi, 2002; Tavassoli et al., 2007; Rasuli et al., 2012; Ghadrdan‐Mashhadi et al., 2013)
					Mule	12.5	(Rasuli et al., 2012)
4	Equine coccidiosis	*Eimeria leuckarti*	Fecal	GIT	Horse	7.68	(Ghahfarrokhi et al., 2014)
					Donkey	7.68	(Ghahfarrokhi et al., 2014)
5	Equine piroplasmosis	*Theileria (Babesia) equi, Babesia caballi*	Blood smears and molecular	Tissues	Donkey	50.94	(Abedi et al., 2015)
					Horse	4.1–96.77	(Sakha, 2007; Davoodi et al., 2010; Arfaei et al., 2013; Malekifard et al., 2014; Hassanpour and Nematollahi, 2014; Habibi et al., 2016; Hosseini et al., 2017)
Nematode							
6	Oxyurosis	*Oxyuris equi*	Fecal, necropsy	GIT	Horse	3.84–26	(Eslami et al., 2005; Hossien et al., 2008; Hosseini et al., 2009; Khosravi et al., 2012; Ghahfarrokhi et al., 2014)
					Donkey	11.53	(Ghahfarrokhi et al., 2014)
7	Strongylosis	*Strongylus vulgaris, Strongylus wquinus, Strongylus edentates*	Fecal, necropsy	GIT	Horse	28.3–34	(Eslami et al., 2005; Hossien et al., 2008; Khosravi et al., 2012; Ali and Yagoob, 2015)
					Donkey	100	(Hosseini et al., 2009; Borji et al., 2014; Tavassoli et al., 2016)
8	Cyathostominosis	*Cyathostomum pathratum, Cylicocyclus elongatus, Cylicostephanus longibursatus, Cylicostephanus goldi, Cylicocyclus nassatus*	Fecal, necropsy	GIT	Donkey	53.3	(Hosseini et al., 2009; Oryan et al., 2015)
					Horse	4–22	(Ali and Yagoob, 2015)
9	Parascariosis	*Parascaris equorum*	Fecal, necropsy	GIT	Horse	10–44	(Eslami et al., 2005; Hossien et al., 2008; Khosravi et al., 2012; Ghahfarrokhi et al., 2014)
					Donkey	3.84–20	(Hosseini et al., 2009; Ghahfarrokhi et al., 2014; Tavassoli et al., 2016]
10	Summer Sores (Cutaneous Habronemosis)	*Habronema muscae, Habronema majus (syn H. microstoma), Draschia (Habronema) megastoma*	Fecal, necropsy	GIT	Donkey	1.72–66.6	(Hosseini et al., 2009; Tavassoli et al., 2016)
11	Lungworm infection	*Dictyocaulus arnfieldi*	Fecal	Lung	Horse	‐	(Sharifi et al., 2010)
12	Probstmayriosis	*Probstmayria vivipara*	Necropsy	GIT	Donkey	20	(Hosseini et al., 2009)
13	Trichostrongylosis	*Trichostrongylus axei*	Necropsy	GIT	Donkey	6.6	(Hosseini et al., 2009)
14	Filariosis	*Setaria equina*	Necropsy	GIT	Donkey	6.6	(Hosseini et al., 2009)
15	Equine parafilariosis	*Parafilaria multipapillosa*	Blood smears	Tissues	Horse and Donkey	1.4–41.3	(Maloufi, 1995)
Trematode							
16	Dicrocoeliosis	*Dicrocoelium denderiticum*	Fecal, necropsy	Intestines	Horse	17.14–56	(Khosravi et al., 2012)
					Donkey	6.6	(Hosseini et al., 2009)
17	Fasciolosis	*Fasciola hepatica*	Necropsy	GIT, Liver	Donkey	6.6	(Hosseini et al., 2009)
Cestode							
18	Anoplocephalosis	*Anoplocephala perfoliata*	Necropsy	GIT	Donkey	12.3	(Hosseini et al., 2009)
19	Equine hydatidosis or echinococcosis	*Echinococcus granulosus*	Necropsy	Liver	Horse, donkey	3.11	(Eslami et al., 2014; Sakhaee et al., 2016;)
Ascari							
20	Tick infestation	*Hyalomma* spp.*, Rhipicephalus (Boophilus)* spp.	Hand picking	Body surfaces	Horse	16.45–52	(Davoodi et al., 2010; Khosravi et al., 2012)
Insect (Parasitic fly)							
21	Gasterophilosis	*Gasterophilus intestinalis, G. nasalis, G. inermis*	Necropsy	Stomach	Horse, Donkey, Mule	16.07–66.6	(Hosseini et al., 2009; Tavassoli and Bakht, 2012; Mashayekhi and Ashtari, 2013)

Abbreviation: GIT, Gastrointestinal tract.
