# Note

**DOI:** 10.1002/vms3.486

**Published:** 2021-06-18

We would like to thank the reader who highlighted the errors in this published review on parasites in horses in Iran for bringing this matter to our attention. The authors have addressed many of their concerns and the correction has been published here. *Veterinary Medicine and Science* encourages post publication discussion and would welcome readers to contact the journal if errors are identified.
